# Degree of kidney injury due to artificial pelvic-ureteric junction obstruction with level of neutrophil gelatinase-associated lipocalin, interleukin 18, and histopathological descriptions in Wistar: experimental research

**DOI:** 10.1097/MS9.0000000000000770

**Published:** 2023-05-03

**Authors:** Yacobda Hamonangan Sigumonrong, Ridha Dharmajaya, Syah Mirsya Warli, Putri Chairani Eyanoer, Irfan Wahyudi, Rosita Juwita Sembiring, Tengku Ibnu Alferraly, Muhammad Rusda

**Affiliations:** Departments of a Urology; b Neurosurgery; c Community and Preventif Medicine; d Obstetrics and Gynecology; e Clinical Pathology; f Anatomical Pathology, Faculty of Medicine, Universitas Sumatera Utara, Medan; g Department of Urology, Faculty of Medicine, Universitas Indonesia, Depok, Indonesia

**Keywords:** Histopathology, IL-18, NGAL, ureteropelvic junction obstruction

## Abstract

Pelvic-ureteric junction obstruction (PUJO) is considered the most common pathology for hydronephrosis in neonates. Full recovery of kidney impairment due to PUJO is possible, especially when pyeloplasty is indicated as surgery is mostly conducted when deterioration is identified, early detection should be considered to prevent further complications. Commonly used kidney damage biomarkers are not sensitive enough to predict kidney damage. Neutrophil gelatinase-associated lipocalin (NGAL) and urinary interleukin 18 (IL-18) are markers of early kidney damage with different characteristics. This study aimed to evaluate the relationship between these two markers with the degree of histopathological kidney damage in Wistar rats induced by PUJO.

**Methods:**

A total of thirty male Wistar rats, 200–250 g, were divided into three groups: (1) control, (2) sham, (3) PUJO (4th, 7th, 14th, and 21st days). Urine NGAL, IL-18 levels, and renal histopathology were observed on day 0, 4, 7, 14, and 21. Statistical analysis was performed using the Kruskal–Wallis and Mann–Whitney test with *P* less than 0.05 considered significant.

**Results:**

There was no significant difference in urine NGAL levels between groups, while IL-18 levels were significantly different based on the Kruskal–Wallis test (*P* 0.031). The results of the Mann–Whitney test showed a significant difference in IL-18 levels between the control group and the PUJO group on day 4 (*P*=0.028); the Sham surgery group with the PUJO group on day 4 (*P*=0.014); the PUJO group on day 4 with the PUJO group on the 7th day (*P*=0.008); and the PUJO group on the 7th day with the PUJO group on the 14th day (*P*=0.033).

**Conclusion:**

Urinary IL-18 levels can be used as a predictor of kidney damage in acute-subacute PUJO cases.

## Introduction

Highlights

- Pelvic-ureteric junction obstruction can lead to kidney failure if not treated immediately, but the operating indications are there only to operate when the damage has been done.
- Commonly used kidney damage biomarkers are not sensitive enough to predict kidney damage.
- Urinary interleukin 18 levels can be used as a predictor of kidney damage in acute-subacute PUJO cases but further studies are needed.

Hydronephrosis is dilatation of the renal pelvis to the renal calyces due to urinary stasis or reflux. Antenatal hydronephrosis is found in about 1–5% of all pregnancies, and 41–88% of antenatal hydronephrosis is temporary or physiological hydronephrosis. The most common pathological hydronephrosis are pelvic-ureteric junction obstruction (PUJO)^1,2^. PUJO is the most common cause of hydronephrosis in neonates. It is estimated that the incidence of this disorder is 1:1500 births and is more common in men and on the left (male: female ratio=2:1)^3^. Compared with developed countries, Indonesia’s detection rate of antenatal hydronephrosis is still relatively low^4,5^. The clinical picture of upper urinary tract obstruction varies greatly depending on the location, degree, and duration of obstruction ranging from low back pain radiating to the lower abdomen and testicles or labia intermittently (50%), asymptomatic abdominal mass, failure to thrive, fever of unknown origin, recurrent urinary tract infections, haematuria, and hypertension^6–8^. In adult patients, the symptoms are minimal, so PUJO is found incidentally^6,8^.

According to the European Association of Urology manual, surgery is indicated if.

1. there is an impairment of the affected kidney below 40%
2. there is a functional decrease of greater than 10% on the affected kidney in subsequent follow-ups;
3. increased anteroposterior diameter; and.
4. kidney dilatation grade III and IV found on ultrasound examination.

Full recovery of kidney impairment due to PUJO is possible, especially when pyeloplasty is indicated. As surgery is mostly conducted when deterioration is identified, early detection should be considered to prevent further complications. Therefore, there is a need for biomarkers that could detect PUJO-associated kidney injury as early as possible. Therefore, surgery and other intervention can be carried out sooner^2,3,8–10^. Hashim and Woodhouse’s study of 142 children with PUJO found 9 children have poor renal function (<20%) and another 27 children with moderate renal function (20–39%)^6^.

Currently, there is no biochemical markers that could predict PUJO-associated kidney injury^3^. Commonly used biochemical markers such as creatinine and urea are less sensitive in the early stages and are strongly influenced by diet and muscle mass^7,11–13^. Even though other markers such as β-trace protein, Inulin, Iohexol, protein- uria, vanin-1, Alpha-glutathione s-transferase, monocyte chemoattractant protein 1, kidney injury molecule 1, *N*-acetyl-*β*-0-glucosaminidase, and liver type fatty acid-binding protein have been widely used in research, they do not provide valuable screening when kidney damage has already occurred. Thus, they are not suitable as predictors for PUJO-associated kidney damage^7,10,14,15^. Radioactive markers also possess an important value to measure kidney function. However, they are not good enough to assess potential risk of impaired renal function^16,17^.

Neutrophil Gelatinase-associated lipocalin (NGAL) is an iron-carrying protein secreted by injured renal tubules. NGAL levels in blood and urine can be used as an early biochemical marker in Acute Kidney Injury (AKI), and a marker for AKI conditions leading to chronic kidney diseases^18–20^. An increase in serum NGAL can be observed as early as 2 h after kidney injury. In a study conducted by Wasilewska in 2011, NGAL was reported to significantly increase the incidence of obstructive nephropathy^21,22^. In one study by Ning *et al*.^23^, it was reported that NGAL had a sensitivity of 70.6% and a specificity of 83.3%. Therefore, urine NGAL levels can be used to detect kidney injury caused by an obstruction such as PUJO.

Interleukin 18 (IL-18) is a pro-inflammatory cytokine that is closely related to the degree of renal fibrosis^24^ and is significantly increased in AKI conditions^25^. Studies have shown that IL-18 increases faster than serum creatinine in AKI (83.8% of patients had an increase in IL-18 value 24 h after kidney injury compared to 6.7% of patients who had an increase in serum creatinine). This linear relationship makes IL-18 often used as a marker to predict the severity of renal ischaemia-reperfusion injury^26^. Measurement of urine IL-18 is easy, fast, and inexpensive, thus facilitating its practicality as a biomarker for patients with AKI.

However, the results of another study showed that the performance of urinary IL-18 levels in the prediction of AKI was less than optimal, with low sensitivity (0.58; 95% CI, 0.52–0.64) and moderate specificity (0.75; 95% CI)., 0.70–0.80)^27^. The study of Liu and colleagues showed that urinary IL-18 levels did not perform well in detecting AKI because IL-18 and inflammation were closely related. IL-18 levels are elevated rapidly in sepsis^28^ and patients with cardiopulmonary bypass or cardiac function problems^7^. These systemic disease factors may attenuate the predictive value of IL-18, as Liu also found better diagnostic accuracy of IL-18 in children and adolescents with fewer comorbidities. Urinary IL-18 levels also change temporally: dramatically increase at 4–6 h, peak at 12 h, and remain elevated up to 48 h after cardiopulmonary bypass. Devarajan demonstrated (in the four phases of experimental AKI: initiation, extension, maintenance, and recovery) that increased IL-18 levels appeared to occur in the second phase, thus responding less rapidly than NGAL levels, which were elevated in the initiation phase^7^.

This study aimed to evaluate the relationship between urinary NGAL and IL-18 levels, two markers of kidney injury that have different characteristics, in experimental animal models with Uretero Pelvic Junction obstruction as a form of kidney injury with microscopic levels of renal fibrosis.

## Methods

### Research design and location

Laboratory experimental using Wistar rats was carried out in November–December 2022. Ethical approval was granted on 7 June 2022. Urine NGAL and IL-18 levels and histopathological features were the dependent variables which measured on their respective observation day (0, 4th, 7th, 14th, or 21st). NGAL and IL-18 levels were measured using the ELISA kit (pg/l), while histopathological features were assessed based on its interstitial inflammation, arterial hyalinosis, interstitial fibrosis, and tubular atrophy. Wistar rats which used in this experiment were preconditioned to obtain homogenous condition among samples. Experimental animals used in this study were male Wistar rats weighed 200–250 g.

### Surgical procedure

**Figure 1:**
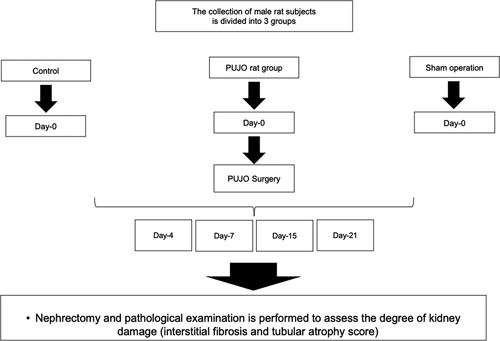
The research procedures schematic. PUJO, pelvic-ureteric junction obstruction.

A total of 36 rats were divided into three groups randomly: (1) control, (2) sham, and PUJO (which is further evaluated and divided into 4th, 7th, 14th, and 21st days). The Abdominal Wall of Wistar rats in control group were not incised at all, while the sham group only underwent laparotomy without ureteral ligation. All of the Wistar rats in PUJO group were underwent ureteral ligation in the same day. However, they were dissected in different time according to their respective group. The schematic of the procedure is shown in Figure 1. This study is reported according to the ARRIVE statement.^29^

1. Perform all procedures with sterile (autoclaved) instruments and consumables.
2. Give anaesthesia to rats with Ketamine+Xylazine, 87.5% and 12.5 injected intraperitoneally.
3. Place the rat on the heated surgical pad in the supine position and secure the limbs to the pad using tape.
4. Incision on flank with knife, cutis, subcutis, muscle is opened layer by layer to access the peritoneal cavity.
5. The kidney and pelvic are identified, carefully separate the ureter from the surrounding tissue.
6. Insert the wing needle into the ureter. Make sure the urine drops out of the tip of the wing needle to ensure it enters the ureteric cavity.
7. Insert prolene thread 6.0.
8. Then, 6.0 vicryl suture is used to ligate the ureter in section proximal to the needle wing insertion site.
9. After the ureter is ligated, the prolene 6.0 thread is removed to create situations of partial obstruction of the ureter.
10. The intra-abdominal organs are put back in place.
11. The wound is closed layer by layer.

### IL-18 testing techniques

1. Prepare all specified reagents, standard solutions, and samples. Before using any reagent, bring it to room temperature. This procedure is carried out at room temperature.
2. Determine the appropriate number of test strips. Use the strip by tucking it in.
3. Add 50 ul to standard well. Do not add antibodies to standard wells, because the standard solution already contains biotinylated antibodies in frame. Unused strips should be stored at 2–8°C.
4. Add 40 ul of sample to the sample well, 10 ul of Mice IL-18 antibody to the sample well, and 50 ul of streptavidin- HRP to the sample well and standard well (Not an empty control well). Mix well. Use sealant to cover the plates. Incubation 60 minutes at 37°C.
5. Remove the sealer and wash the dishes thoroughly five times with wash buffer. Soak the wells in 300 ul wash buffer for thirty seconds to 1 min per wash. For automatic washing, suck or pour each well and wash with wash buffer five times. Dishes should be cleaned with paper towels or other absorbent materials.
6. Each well should receive 50 ul of substrate solution A, followed by 50 ul of substrate solution B. At 37°C and in the dark, incubate the plate with the new sealant for 10 min.
7. Add 50 ul Stop Solution to each well, and the blue colour will immediately change to yellow.
8. Determine the optical density (OD value) of each well using a microplate reader calibrated to 450 nm within 10 min of applying the stopping solution.

### Lipocalin test technique for neutrophil gelatinase

1. Prepare all specified reagents, standard solutions, and samples. Before using any reagent, bring it to room temperature. This procedure is carried out at room temperature.
2. Determine the appropriate number of test strips. Use the strip by inserting it into the frame. Unused strips should be stored at 2–8°C.
3. Add 50 ul to standard well. Do not add antibodies to the standard well because the standard solution already contains biotinylated antibodies.
4. Add 40 ul of sample to sample well, 10 ul of Mouse LCN2 antibody to sample well, and 50 ul of streptavidin-HRP to sample well and standard well (Not empty control well). Mix well. Use sealant to cover the plates. Incubation 60 min at 37°C.
5. Remove the sealer and wash the dishes thoroughly five times with wash buffer. Soak the wells in 300 ul wash buffer for 30 s to 1 min per wash. For automatic washing, suck or pour each well and wash with wash buffer five times. Dishes should be cleaned with paper towels or other absorbent materials.
6. Each well should receive 50 ul of substrate solution A, followed by 50 ul of substrate solution B. At 37°C and in the dark, incubate the plate with the new sealant for 10 min.
7. Add 50 ul Stop Solution to each well, and a blue colour will appear immediately turned yellow.
8. Determine the optical density (OD value) of each well using a microplate reader calibrated to 450 nm within 10 min of applying the stopping solution.

### Histopathological preparations

1. Kidney tissue is fixed with 10% formalin.
2. The tissue is sliced with a thickness of 3–5 mm and inserted into the tissue cassette.
3. The tissue is dehydrated by immersing it in a liquid, such as alcohol, toluene, xylol, and liquid paraffin.
4. Do embedding and blocking on the sample network.
5. Then cut using a microtome with a thickness of 4–6 µm, close to a 56°C water bath, for tissue development sites. If it has expanded, catch the tissue with a glass object and dry it deep room temperature. Then put it in the incubator before staining 6. Then stain the preparation and immerse it in the solution haematoxylin for 15 min.
6. After all the staining processes have been completed, then dry and ready to read.

### Statistical method

Univariate analysis was performed to obtain the distribution of sample characteristics. Data analysis is then presented in frequency distribution for categorical data, mean ± SD for normally distributed numerical data, and median (minimum–maximum) for non-normally distributed numerical data. For data analysis, ANOVA was used for normally distributed data and the Kruskal–Wallis test for data that were not normally distributed. Analysis of the data will be carried out using statistical software (SPSS 21).

## Results

**Figure 2:**
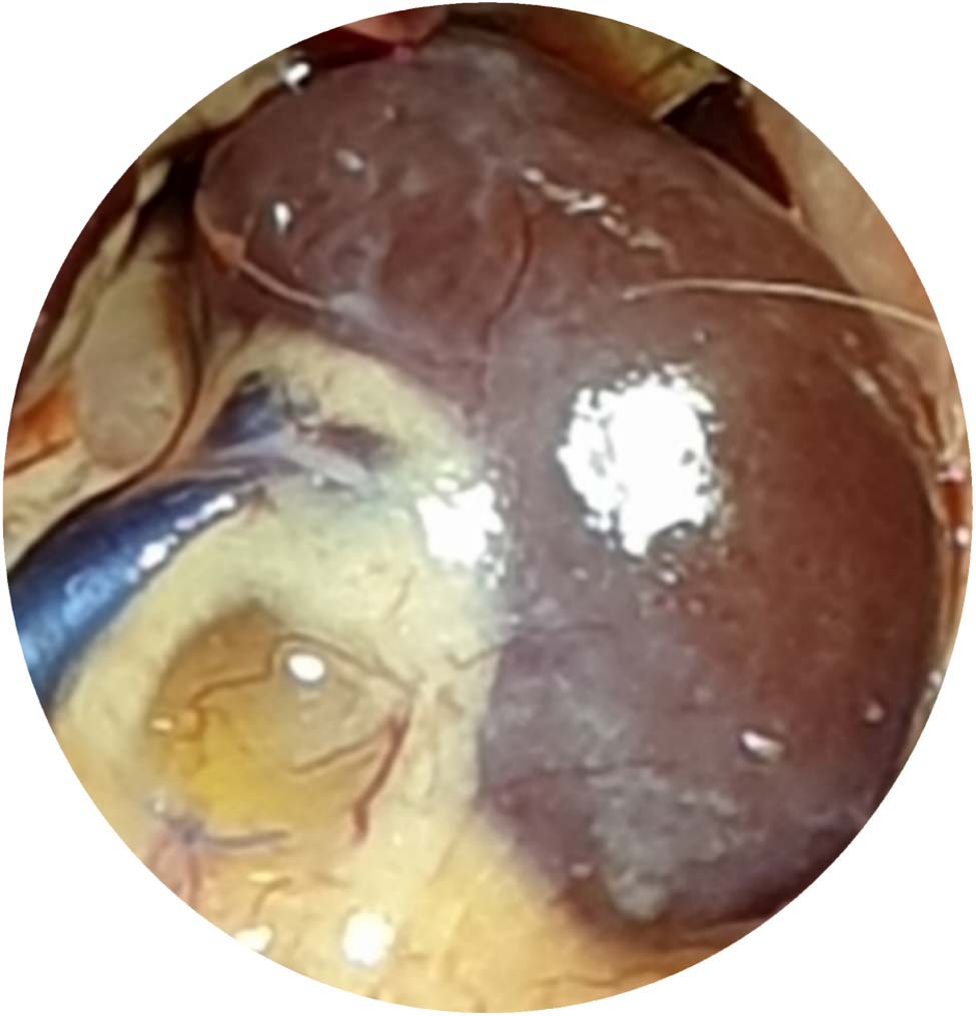
Macroscopic appearance of PUJO kidney. PUJO, pelvic-ureteric junction obstruction.

**Figure 3:**
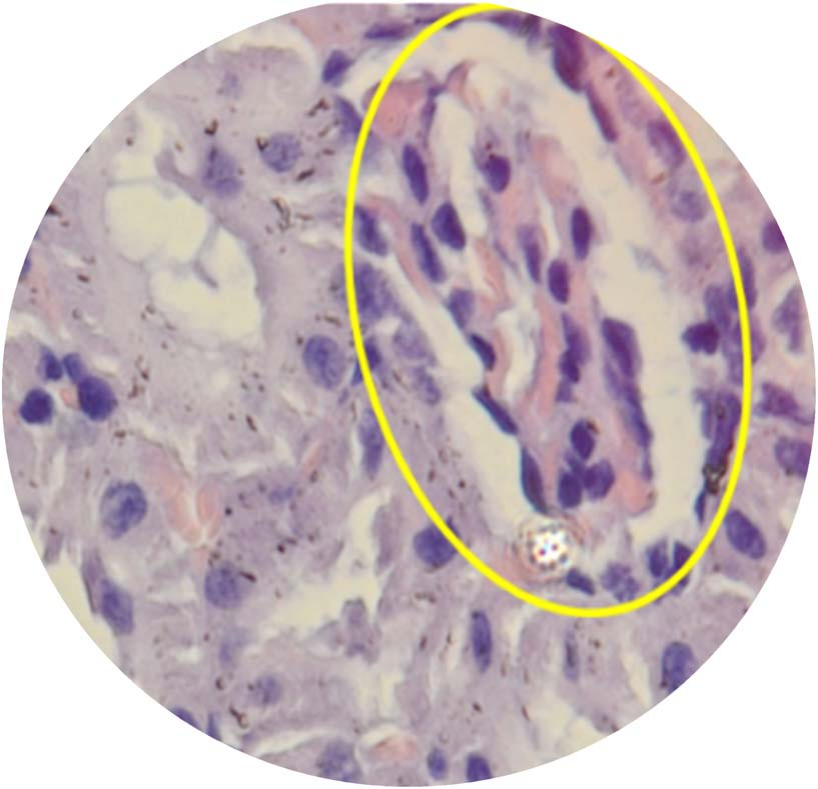
Glomerulosclerosis.

The highest mean of urine NGAL level were found in the PUJO day 7th group, while the lowest were found in the control group. Based on the Kruskal–Wallis test, there was no significant difference in NGAL values between groups (*P*=0.063). Although the mean difference did not show significant results, there was a progressive increase in NGAL starting from day 4 and reached the peak on day 7 which then decreased on day 14 (Table 1). On the other hand, the urine IL-18 levels between the PUJO day 4th, PUJO day 7th, PUJO day 14th, PUJO day 21th, control, and sham groups were significantly different based on the Kruskal–Wallis test (*P*=0.031), as shown in Table 2. The results of the Mann–Whitney test showed that there was a significant difference in IL-18 levels between the control group and the PUJO group day 4th (*P*=0.028). There is also marked difference between sham surgery group and the PUJO group day 4th (*P*=0.014), sham surgery and the PUJO group day 7th and the sham surgery and the PUJO group day 14th. There is also significant difference between PUJO group day 4th and PUJO group day 7th (*P*=0.008). PUJO group day 14th also showed obvious differences (*P*=0.033). The relationship between groups in detail is shown in Table 3. This research rely on how the operation procedure was performed. We are not only assessed gross appearance of the kidney, but we also conducted histopathological analysis to confirmed whether the ligation on the pelvic-ureteric junction managed to create a true PUJO model or not (Fig. 2). The result was expected. The kidney suffered a damage in various form such as Glomerulosclerosis, Interstitial Inflammation, Interstitial Fibrosis, and Tubular Atrophy (Fig. 3 – Fig. 6).

**Table 1:** NGAL mean value in each group (Kruskal–Wallis Test)

Group	Mean+SD	Median (Min–Max)	*P*
Control	14.89±4.66	13.11 (10.31–24.35)	
Sham	15.40±4.80	15.96 (7.77–21.60)	
4 days	17.02±5.18	15.62 (11.17–26.58)	0.063
7 days	21.24±7 36	19.11 (14.25–37.04)	
14 days	18.42±3.33	18.17 (14.32–22.73)	
21 days	16.12±2.98	16.48 (10.16–20.34)	

Max, maximum; Min, minimum; NGAL, neutrophil gelatinase-associated lipocalin.

**Table 2:** The mean value of IL-18 levels in each group (Kruskal–Wallis test)

Group	Mean+SD	Median (Min–Max)	*P*
Control	83.37+29.56	89.40 (33.03–95.54)	
Sham	89.40+33.03	85.68 (49.38–145.41)	
4 days	107.37+18.37	102.87 (73.84–135.86)	0.031^*^
7 days	110.05+19.86	99.89 (90.57–147.53)	
14 days	116.88+21.44	119.24 (79.48–148.73)	
21 days	94.82+35.13	91.80 (48.44–163.88)	

IL-18, interleukin 18; Max, maximum; Min, minimum.

* *P*<0,05 is considered significant.

**Table 3:** Results of post hoc analysis for IL-18 levels (Mann–Whitney Test)

	Control	Sham	4 days	7 days	14 days	21 days
Control	—	0.977	0.028^*^	0.266	0.143	0.178
Sham	—	—	0.014^*^	0.443	0.160	0.178
4 days	—	—	—	0.008^*^	0.713	0.319
7 days	—	—	—	—	0.033^*^	0.089
14 days	—	—	—	—	—	0.932

IL-18, interleukin 18.

* *P*<0,05 is considered significant.

**Figure 4:**
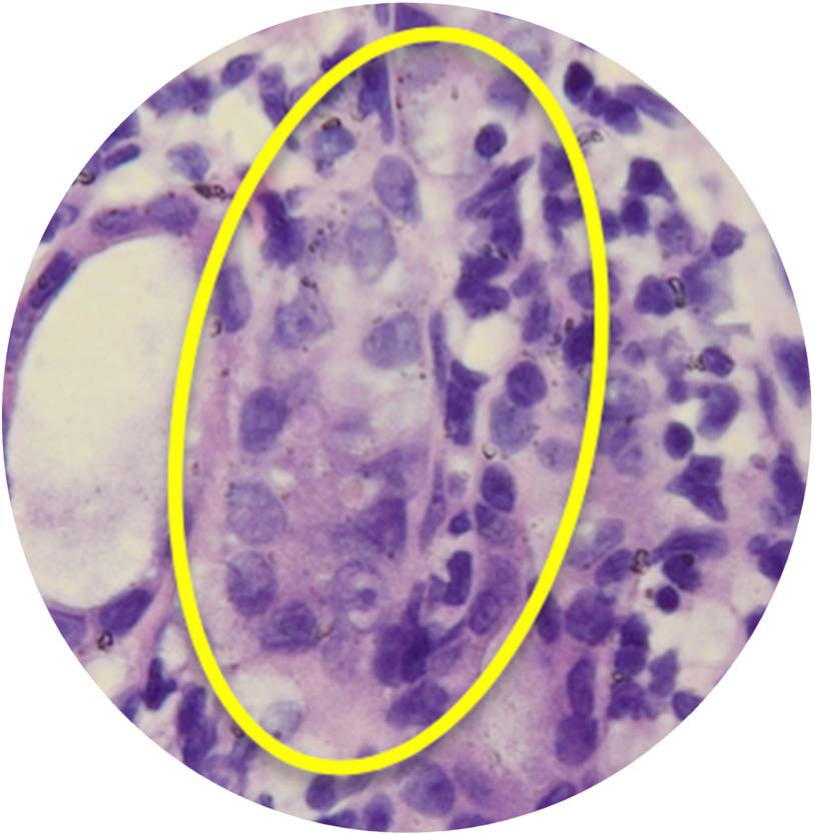
Interstitial inflamation.

**Figure 5:**
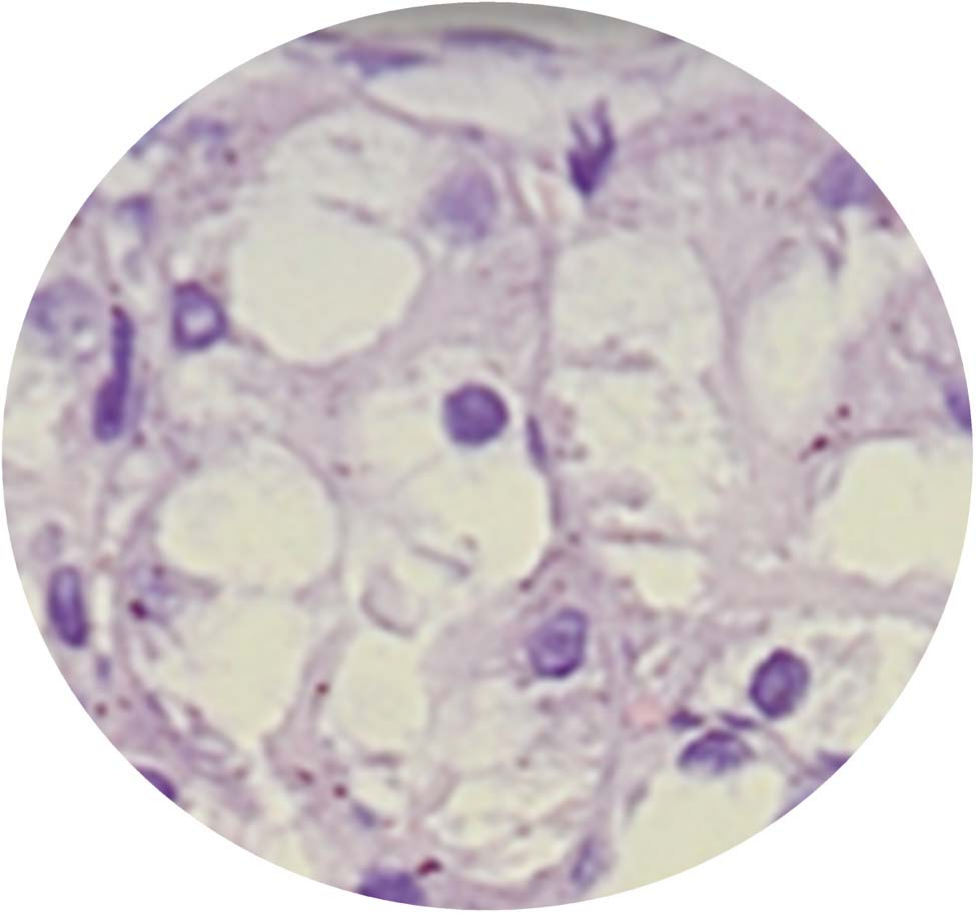
Tubular atrophy.

**Figure 6:**
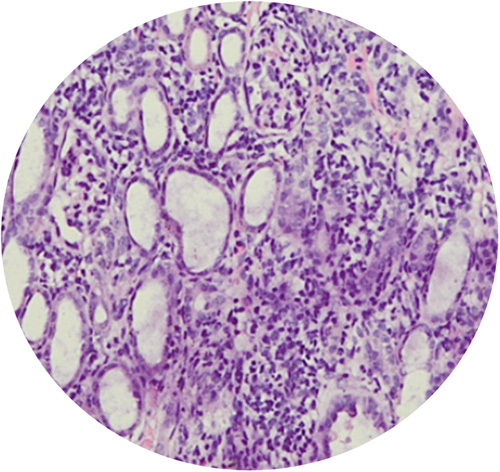
Interstitial fibrosis.

**Figure 7:**
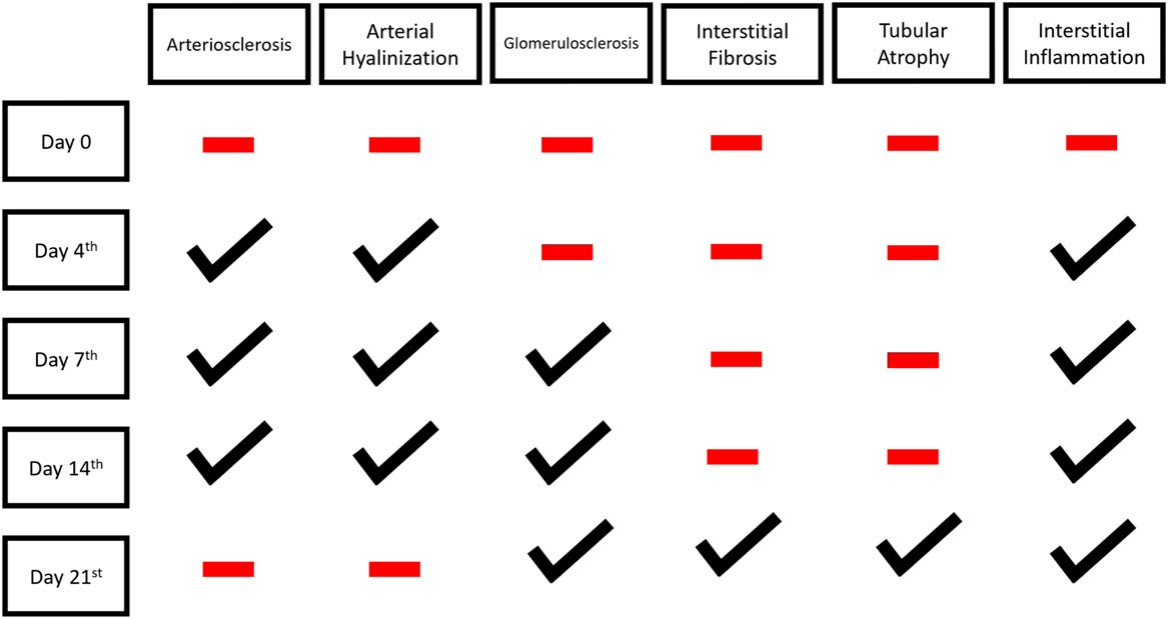
Timeline of histological features occurence.

In this study, we conducted histopathological examination with HE Staining. We used several parameters to determine severity and progression of the kidney injury, which are arteriosclerosis, arterial hyalinization, glomerulosclerosis, interstitial fibrosis, tubular atrophy, and interstitial inflammation. As depicted in the Figure 7, arteriosclerosis, arterial hyalinization, and interstitial inflammation occurred earlier. It started to appear in day 4th. Those processes then followed by Glomerulosclerosis which occurred on the day 7th. interstitial fibrosis and tubular atrophy did not occur until day 21st.

## Discussion

Serum creatinine and urine volume are commonly used to measure kidney damage^30^. However, ureum and creatinine were known to increased only in already injured kidney. There is an unmet demand of biomarker which could detect early kidney damage. Kidney injuries could increase levels of various biomarkers. NGAL and IL-18 are two emerging novel biomarker which hailed as biomarker which has high sensitivity and specificity toward early kidney injury. Theoretically, NGAL could increase in the event of the death of renal tubular cells. Increased intrarenal pressure due to obstruction can result in renal tubular cell death. Indirectly, increased intrarenal pressure will increase angiotensin II, causing renal ischaemia and necrosis of renal tubular cells^31^. NGAL is known to be detectable in urine within 2 h of renal ischaemia^32^.

Meanwhile, IL-18 will increase if there is renal fibrosis. Increased intrarenal pressure will cause an increase in ROS. Increased ROS will cause mesenchymal-epithelial transition, thereby increasing the amount of renal tissue undergoing fibrosis^33^. As previously mentioned, one study showed that most patients had an increase in IL-18 24 h after kidney injury (83.8%). This is in stark contrast to the increase in serum creatinine 24 h after kidney injury, which only occurred in 6.7% of patients in the study^34^.

This study showed significant differences in IL-18 levels between the intervention groups, namely between the control group and the PUJO group on the day 4th, the sham surgery group and the PUJO group on the day 4th, the PUJO group on the day 4th with PUJO group on the day 7th, and PUJO group day 7th with day 14th. Meanwhile, the intervention groups had no significant difference in NGAL levels. Nevertheless, interesting findings show a progressive increase in NGAL from the intervention until day 7th.

From the results of this study, it was found that there was no significant difference between the intervention groups regarding the increase in NGAL levels. This finding is inconsistent with the theory that an increase in NGAL can be detected in the urine within 2 h after kidney injury^32^. Although NGAL has often been studied as a biomarker that can be used to detect kidney damage, it should be noted that NGAL is produced in the kidneys and can also be found in the respiratory tract, stomach, and intestines. In addition, NGAL is not only increased in conditions of kidney damage but can also be increased in conditions of bacterial infection, non-infectious systemic inflammatory response syndrome, and other chronic or systemic diseases. Therefore, it is necessary to consider other causes besides kidney damage in conditions of increased NGAL levels^21^. An increase in nonspecific NGAL caused by kidney damage is one of the shortcomings of NGAL as a biomarker reference to assess kidney damage, including those caused by obstruction^22^.

On the other hand, this study showed significant differences in IL-18 levels between the intervention groups. This finding follows the theory that IL-18 will be increased in fibrotic kidney conditions.^32^. This study’s data showed that the highest mean and median levels of IL-18 were found in the PUJO group on the day 14th. One study reported an increase in IL-18 in patients who developed AKI after undergoing cardiopulmonary bypass surgery^24^. Further research is needed on the duration of the increase in IL-18 in various conditions of kidney damage, including those caused by kidney obstruction. The data obtained from this study indicate that IL-18 can be a more reliable biomarker in predicting kidney damage caused by renal obstruction than the NGAL biomarker. This finding is in line with a study that found there was a strong association between IL-18 levels and the severity of renal fibrosis^24^ analysis for IL-18 levels (Mann–Whitney Test).

The highest average and median values were found in the PUJO group on day 14th, while the lowest average and median values were found in the control group. Based on the Kruskal–Wallis test, there was no significant difference in NGAL values between groups (*P*=0.063). Although the mean difference did not show significant results, there was a progressive increase in IL-18 starting from day 4th and reaching a culmination point on day 7th which then dropped on day 14th. NGAL levels in the PUJO group, in detail, can be seen in Table 1. While the urine IL-18 levels between the PUJO, control, and sham groups were significantly different based on the Kruskal–Wallis test (*P*=0.031), as shown in Table 2. The results of the Mann–Whitney test showed that there was a significant difference in IL-18 levels between the control group and the PUJO group on the day 4th (*P*=0.028), the Sham surgery group and the PUJO group on the day 4th (*P*=0.014), the PUJO group on the day 4th and the PUJO group on the day 4th. 7th day (*P*=0.008), the day 7th PUJO group, and the day 14th PUJO group (*P*=0.033). The relationship between groups in detail is shown in Table 3.

In this study, we conducted a histopathological examination with several parameter to determine the degree of kidney injury. We found an interesting pattern which different from the study which conducted by Jackson *et al.* in 2016^35^. It was stated on the study that in patient with Complete Unilateral Ureteral Obstruction, Tubular Atrophy occurred on day 5th, which then followed by Interstitial Fibrosis in day 7th. Those two occurred on the first week after the injury. On the other hand, this study found that tubular atrophy and interstitial fibrosis started to appear in day 21st. Phenomenon such as arteriosclerosis, arterial hyalinization, interstitial inflammation, and glomerulosclerosis were occurred consecutively prior to tubular atrophy and interstitial fibrosis. The difference between this study and the study conducted by Jackson and colleagues is in this study, the ureter was partially ligated whereas the other study completely ligated the ureter. Thus, we could conclude that kidney injury process in partial ureteral ligated cases is slower compared with total ureteral ligated one. In partial ureteral ligation cases, we could also observe the sequence of kidney injury process in detail. In conclusion, IL-18 could rise really early in kidney injury which caused by unilateral partial obstruction due to arterial hyalinization, arteriosclerosis, and interstitial inflammation processes. Although NGAL levels were not significantly different between the control, sham, and PUJO groups, an increase up to day 7th indicates the predictive value of NGAL in this condition. Urine IL-18 levels can be used as a predictor of kidney damage in early PUJO cases (acute stage). The increase of IL-18 is due to arterial hyalinization, arteriosclerosis and interstitial inflammation processes which occurred as early as day 4th after unilateral ureter was partially ligated. Further studies are needed to assess the predictive accuracy of NGAL and IL-18 prospectively on the incidence of PUJO in human.

## Ethical approval

Ethical approval was granted by the ethical committee of Sumatra Utara University with an ethical number of 496/KEPK/USU/2022.

## Consent

None declared.

## Source of funding

None declared.

## Conflicts of interest disclosure

None declared.

## Provenance and Peer Review

Not commissioned, externally peer-reviewed.
